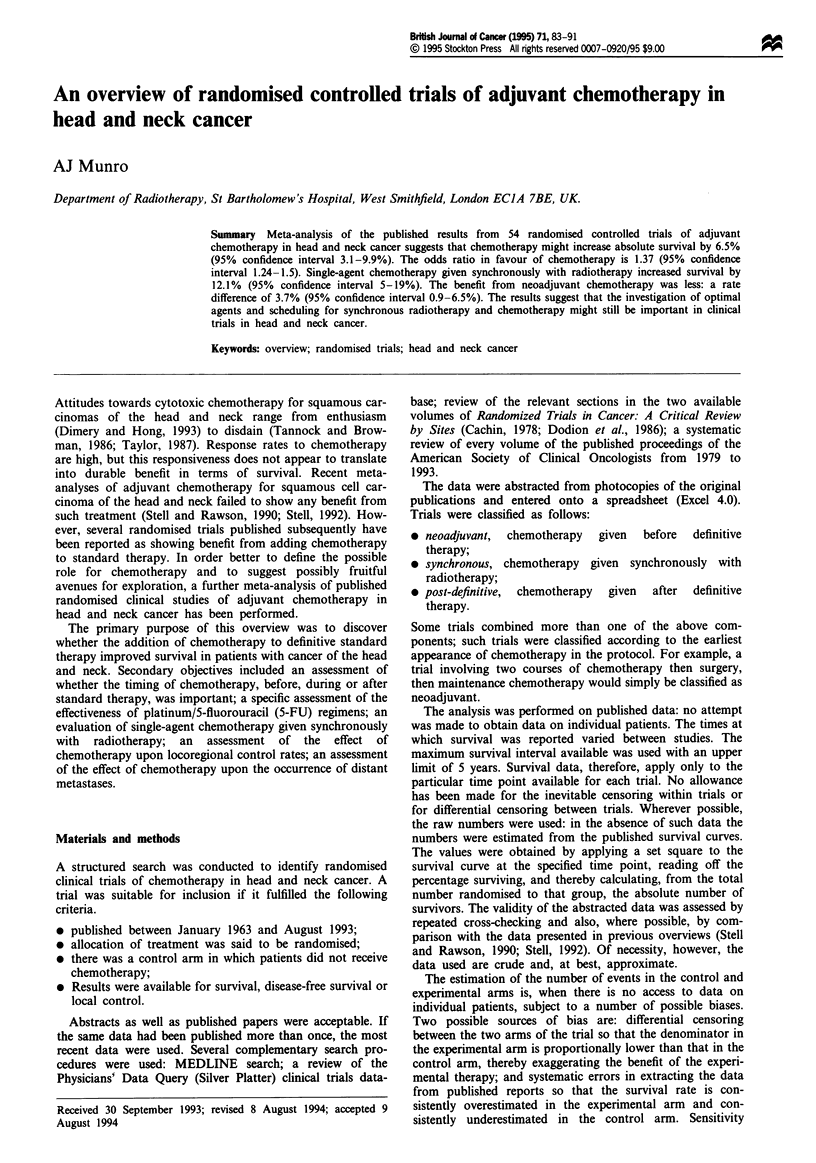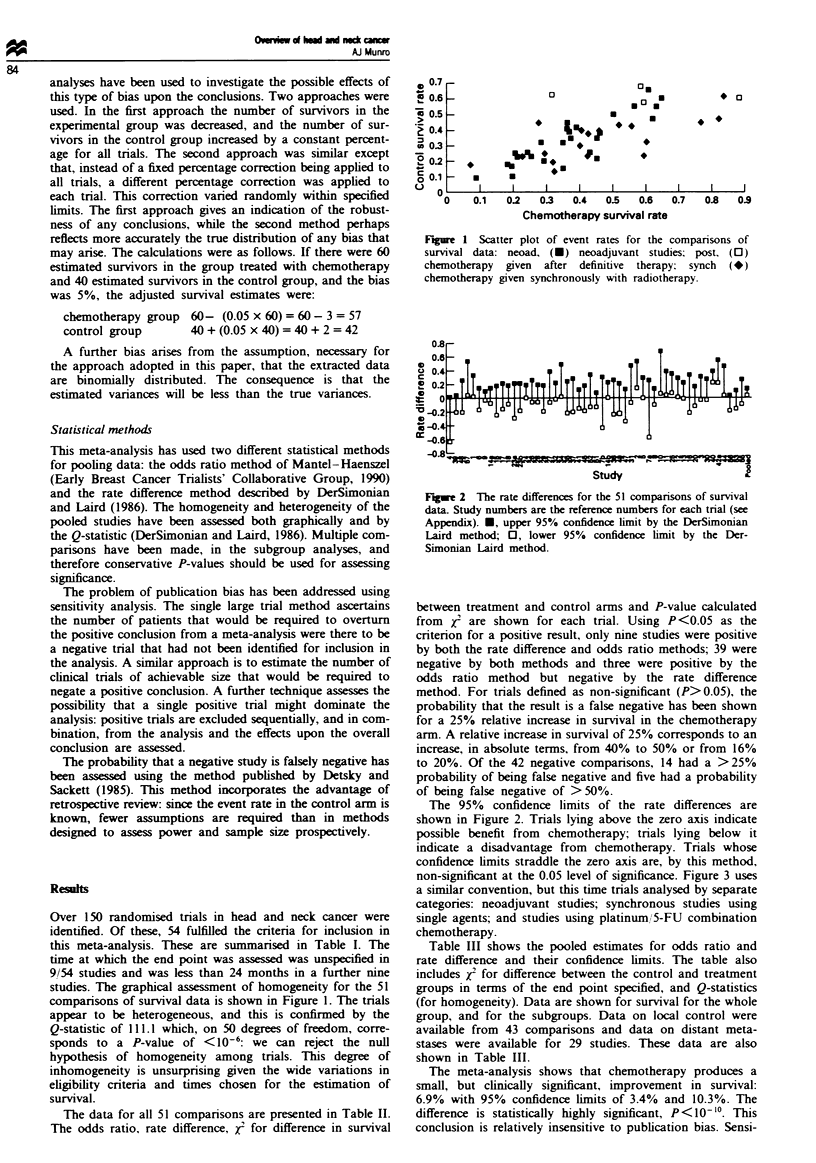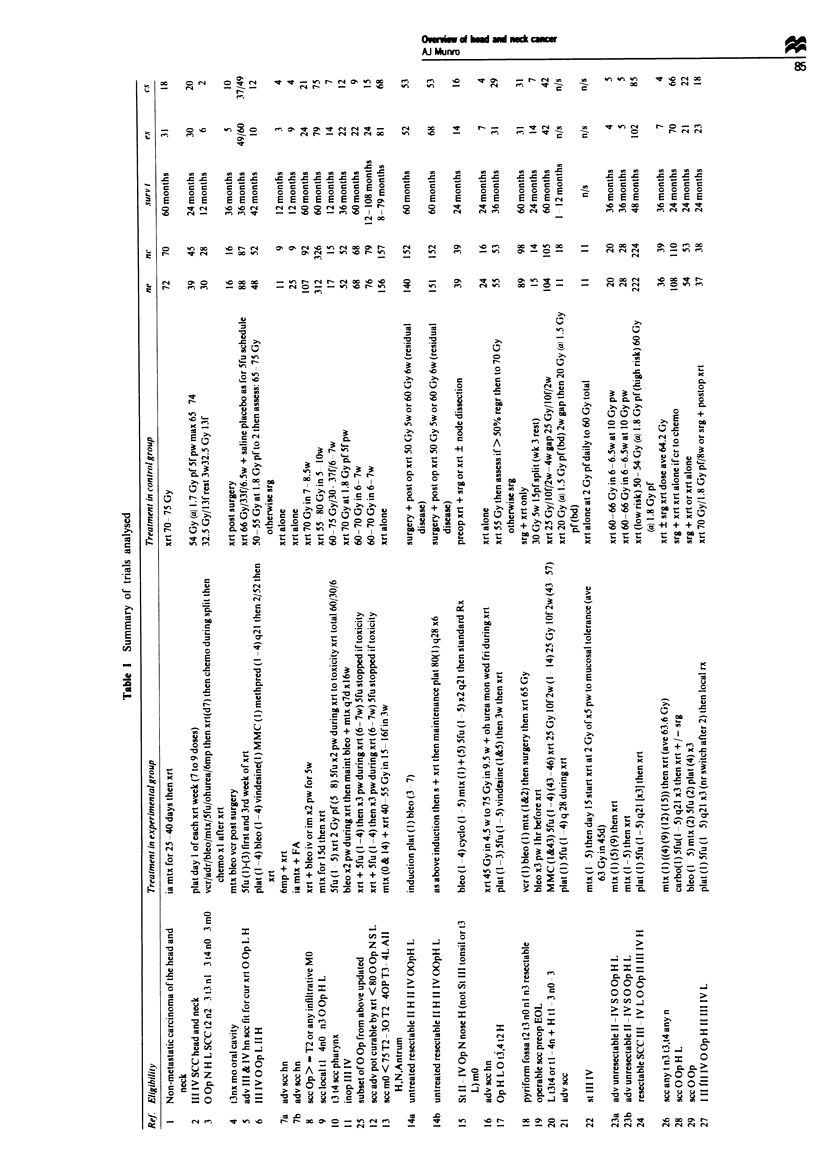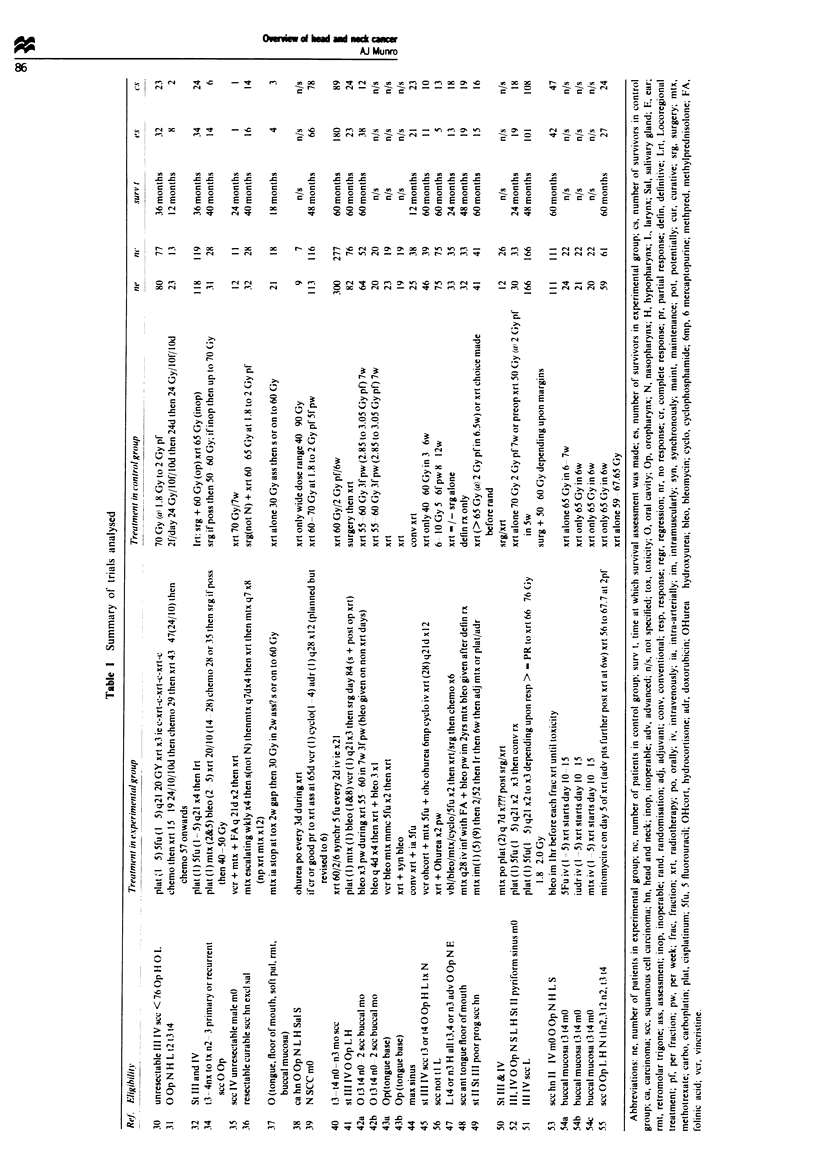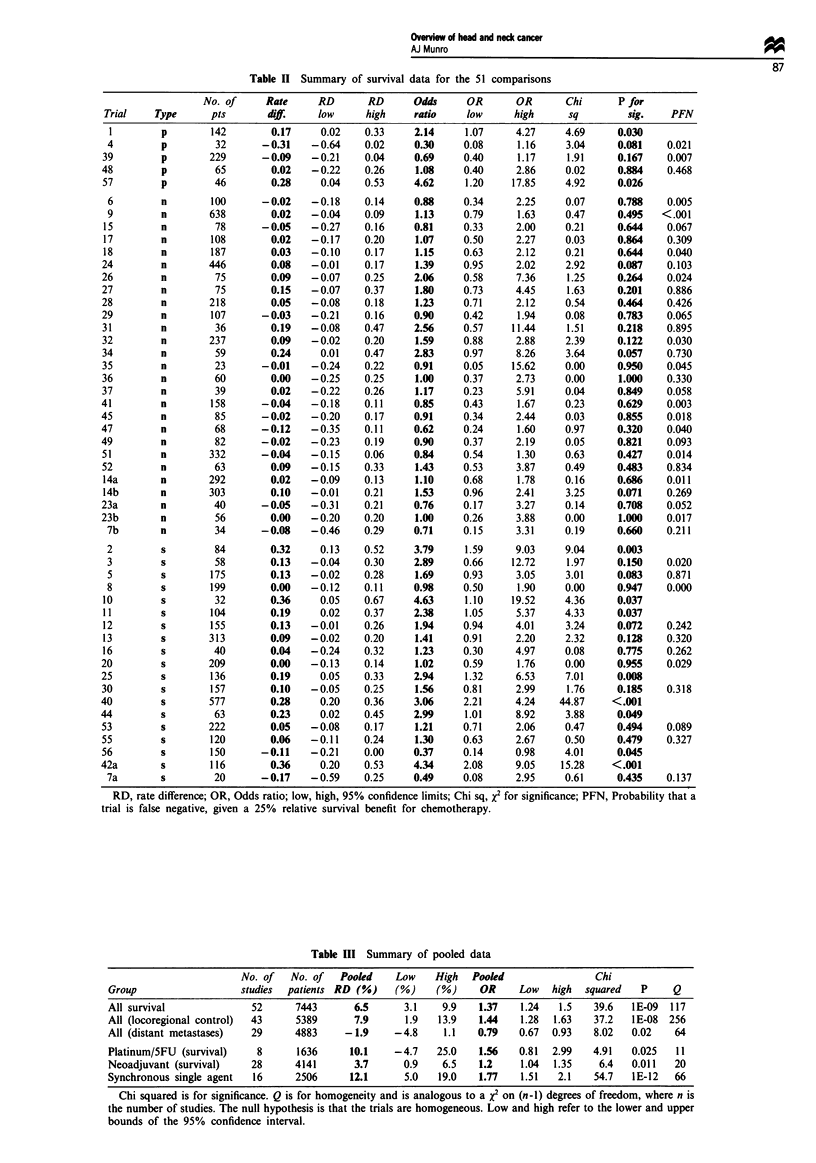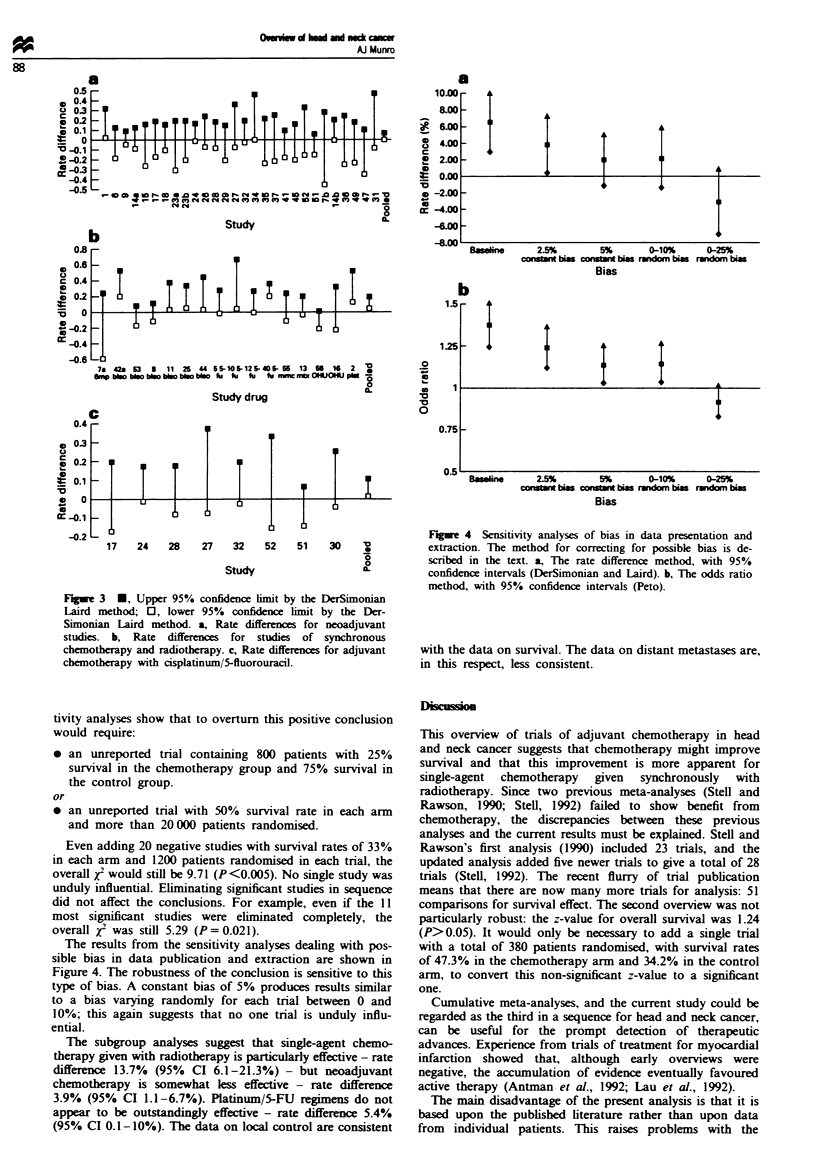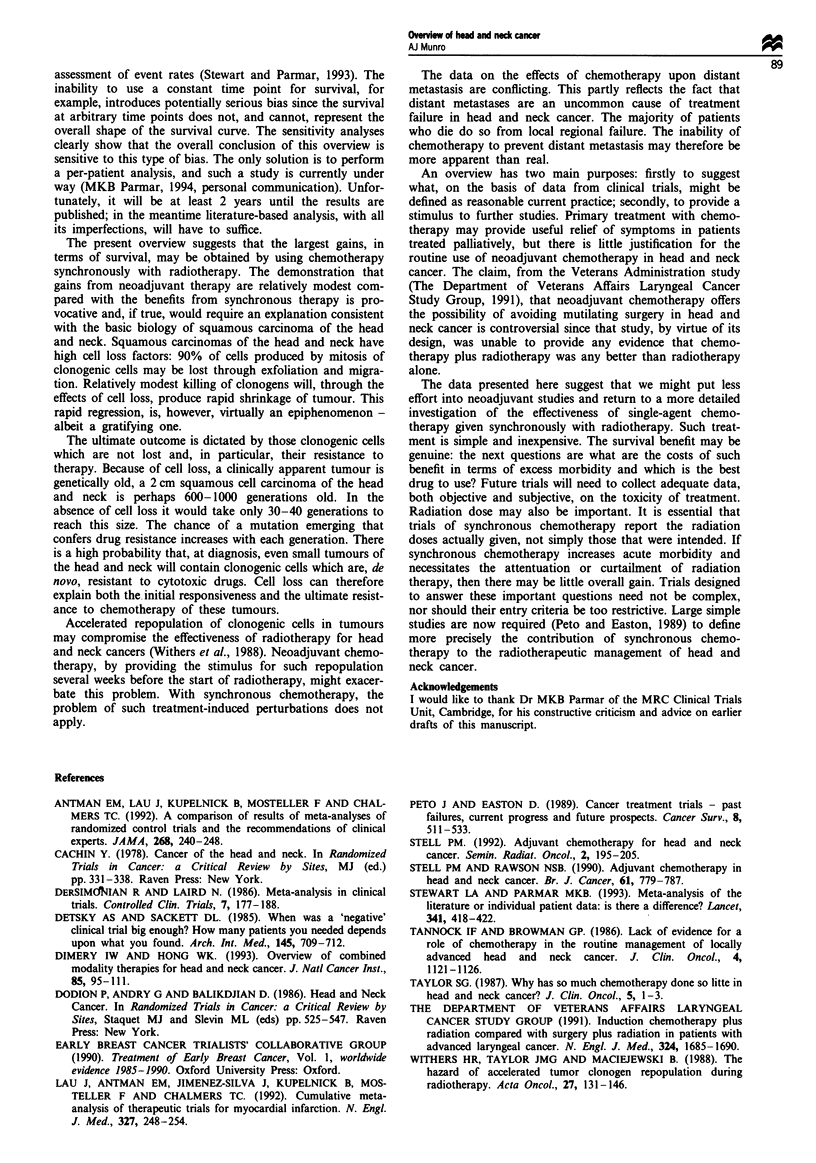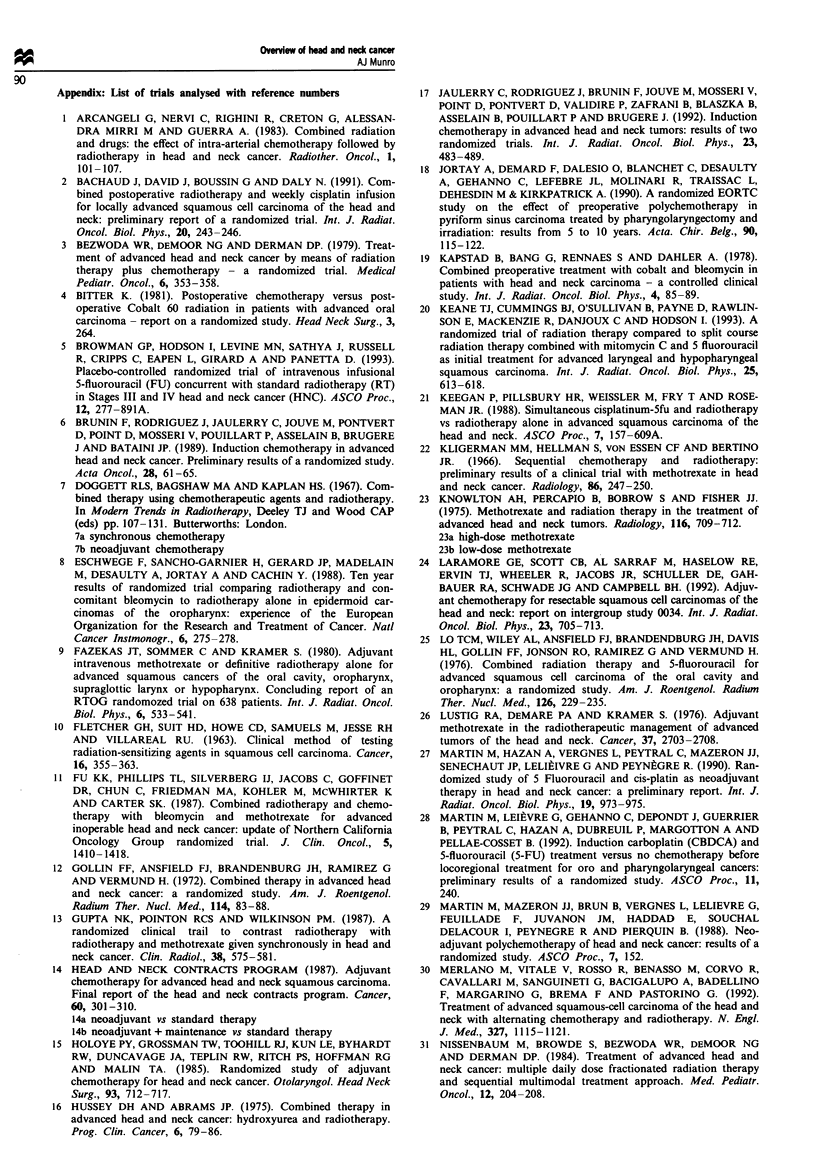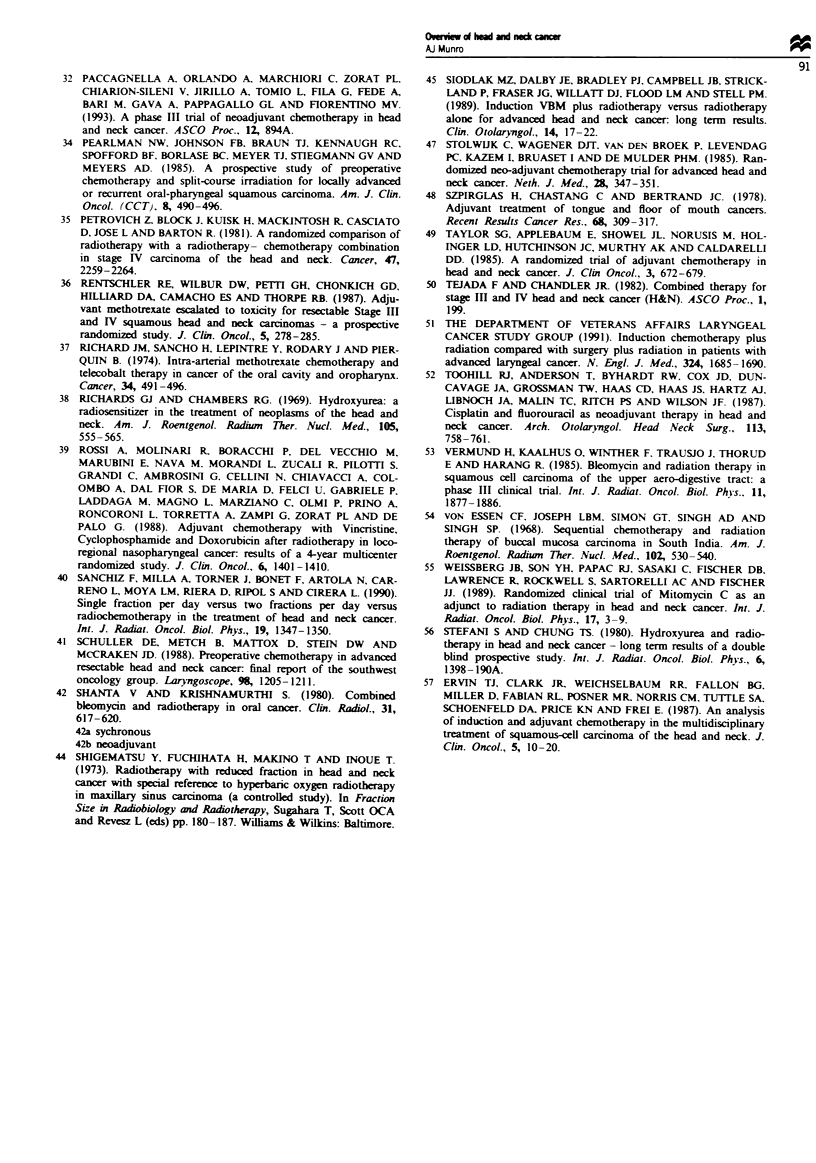# An overview of randomised controlled trials of adjuvant chemotherapy in head and neck cancer.

**DOI:** 10.1038/bjc.1995.17

**Published:** 1995-01

**Authors:** A. J. Munro

**Affiliations:** Department of Radiotherapy, St Bartholomew's Hospital, West Smithfield, London, UK.

## Abstract

Meta-analysis of the published results from 54 randomised controlled trials of adjuvant chemotherapy in head and neck cancer suggests that chemotherapy might increase absolute survival by 6.5% (95% confidence interval 3.1-9.9%). The odds ratio in favour of chemotherapy is 1.37 (95% confidence interval 1.24-1.5). Single-agent chemotherapy given synchronously with radiotherapy increased survival by 12.1% (95% confidence interval 5-19%). The benefit from neoadjuvant chemotherapy was less: a rate difference of 3.7% (95% confidence interval 0.9-6.5%). The results suggest that the investigation of optimal agents and scheduling for synchronous radiotherapy and chemotherapy might still be important in clinical trials in head and neck cancer.


British Journal of Cancer (1995) 71, 83–91
© 1995 Stockton Press All rights reserved 0007-0920/95 $9.00

### An overview of randomised controlled trials of adjuvant chemotherapy in head and neck cancer

AJ Munro

*Department of Radiotherapy, St Bartholomew's Hospital, West Smithfield, London EC1A 7BE, UK.*

**Summary** Meta-analysis of the published results from 54 randomised controlled trials of adjuvant chemotherapy in head and neck cancer suggests that chemotherapy might increase absolute survival by 6.5% (95% confidence interval 3.1–9.9%). The odds ratio in favour of chemotherapy is 1.37 (95% confidence interval 1.24–1.5). Single-agent chemotherapy given synchronously with radiotherapy increased survival by 12.1% (95% confidence interval 5–19%). The benefit from neoadjuvant chemotherapy was less: a rate difference of 3.7% (95% confidence interval 0.9–6.5%). The results suggest that the investigation of optimal agents and scheduling for synchronous radiotherapy and chemotherapy might still be important in clinical trials in head and neck cancer.

**Keywords:** overview; randomised trials; head and neck cancer

Attitudes towards cytotoxic chemotherapy for squamous carcinomas of the head and neck range from enthusiasm (Dimery and Hong, 1993) to disdain (Tannock and Browman, 1986; Taylor, 1987). Response rates to chemotherapy are high, but this responsiveness does not appear to translate into durable benefit in terms of survival. Recent meta-analyses of adjuvant chemotherapy for squamous cell carcinoma of the head and neck failed to show any benefit from such treatment (Stell and Rawson, 1990; Stell, 1992). However, several randomised trials published subsequently have been reported as showing benefit from adding chemotherapy to standard therapy. In order better to define the possible role for chemotherapy and to suggest possibly fruitful avenues for exploration, a further meta-analysis of published randomised clinical studies of adjuvant chemotherapy in head and neck cancer has been performed.

The primary purpose of this overview was to discover whether the addition of chemotherapy to definitive standard therapy improved survival in patients with cancer of the head and neck. Secondary objectives included an assessment of whether the timing of chemotherapy, before, during or after standard therapy, was important; a specific assessment of the effectiveness of platinum/5-fluorouracil (5-FU) regimens; an evaluation of single-agent chemotherapy given synchronously with radiotherapy; an assessment of the effect of chemotherapy upon locoregional control rates; an assessment of the effect of chemotherapy upon the occurrence of distant metastases.

### Materials and methods

A structured search was conducted to identify randomised clinical trials of chemotherapy in head and neck cancer. A trial was suitable for inclusion if it fulfilled the following criteria.

- published between January 1963 and August 1993;
- allocation of treatment was said to be randomised;
- there was a control arm in which patients did not receive chemotherapy;
- Results were available for survival, disease-free survival or local control.

Abstracts as well as published papers were acceptable. If the same data had been published more than once, the most recent data were used. Several complementary search procedures were used: MEDLINE search; a review of the Physicians' Data Query (Silver Platter) clinical trials database; review of the relevant sections in the two available volumes of *Randomized Trials in Cancer: A Critical Review by Sites* (Cachin, 1978; Dodion *et al.*, 1986); a systematic review of every volume of the published proceedings of the American Society of Clinical Oncologists from 1979 to 1993.

The data were abstracted from photocopies of the original publications and entered onto a spreadsheet (Excel 4.0). Trials were classified as follows:

- *neoadjuvant*, chemotherapy given before definitive therapy;
- *synchronous*, chemotherapy given synchronously with radiotherapy;
- *post-definitive*, chemotherapy given after definitive therapy.

Some trials combined more than one of the above components; such trials were classified according to the earliest appearance of chemotherapy in the protocol. For example, a trial involving two courses of chemotherapy then surgery, then maintenance chemotherapy would simply be classified as neoadjuvant.

The analysis was performed on published data: no attempt was made to obtain data on individual patients. The times at which survival was reported varied between studies. The maximum survival interval available was used with an upper limit of 5 years. Survival data, therefore, apply only to the particular time point available for each trial. No allowance has been made for the inevitable censoring within trials or for differential censoring between trials. Wherever possible, the raw numbers were used: in the absence of such data the numbers were estimated from the published survival curves. The values were obtained by applying a set square to the survival curve at the specified time point, reading off the percentage surviving, and thereby calculating, from the total number randomised to that group, the absolute number of survivors. The validity of the abstracted data was assessed by repeated cross-checking and also, where possible, by comparison with the data presented in previous overviews (Stell and Rawson, 1990; Stell, 1992). Of necessity, however, the data used are crude and, at best, approximate.

The estimation of the number of events in the control and experimental arms is, when there is no access to data on individual patients, subject to a number of possible biases. Two possible sources of bias are: differential censoring between the two arms of the trial so that the denominator in the experimental arm is proportionally lower than that in the control arm, thereby exaggerating the benefit of the experimental therapy; and systematic errors in extracting the data from published reports so that the survival rate is consistently overestimated in the experimental arm and consistently underestimated in the control arm. Sensitivity

Received 30 September 1993; revised 8 August 1994; accepted 9 August 1994

analyses have been used to investigate the possible effects of this type of bias upon the conclusions. Two approaches were used. In the first approach the number of survivors in the experimental group was decreased, and the number of survivors in the control group increased by a constant percentage for all trials. The second approach was similar except that, instead of a fixed percentage correction being applied to all trials, a different percentage correction was applied to each trial. This correction varied randomly within specified limits. The first approach gives an indication of the robustness of any conclusions, while the second method perhaps reflects more accurately the true distribution of any bias that may arise. The calculations were as follows. If there were 60 estimated survivors in the group treated with chemotherapy and 40 estimated survivors in the control group, and the bias was 5%, the adjusted survival estimates were:

chemotherapy group $60 - (0.05 \times 60) = 60 - 3 = 57$

control group $40 + (0.05 \times 40) = 40 + 2 = 42$

A further bias arises from the assumption, necessary for the approach adopted in this paper, that the extracted data are binomially distributed. The consequence is that the estimated variances will be less than the true variances.

### *Statistical methods*

This meta-analysis has used two different statistical methods for pooling data: the odds ratio method of Mantel-Haenszel (Early Breast Cancer Trialists' Collaborative Group, 1990) and the rate difference method described by DerSimonian and Laird (1986). The homogeneity and heterogeneity of the pooled studies have been assessed both graphically and by the *Q*-statistic (DerSimonian and Laird, 1986). Multiple comparisons have been made, in the subgroup analyses, and therefore conservative *P*-values should be used for assessing significance.

The problem of publication bias has been addressed using sensitivity analysis. The single large trial method ascertains the number of patients that would be required to overturn the positive conclusion from a meta-analysis were there to be a negative trial that had not been identified for inclusion in the analysis. A similar approach is to estimate the number of clinical trials of achievable size that would be required to negate a positive conclusion. A further technique assesses the possibility that a single positive trial might dominate the analysis: positive trials are excluded sequentially, and in combination, from the analysis and the effects upon the overall conclusion are assessed.

The probability that a negative study is falsely negative has been assessed using the method published by Detsky and Sackett (1985). This method incorporates the advantage of retrospective review: since the event rate in the control arm is known, fewer assumptions are required than in methods designed to assess power and sample size prospectively.

### Results

Over 150 randomised trials in head and neck cancer were identified. Of these, 54 fulfilled the criteria for inclusion in this meta-analysis. These are summarised in Table I. The time at which the end point was assessed was unspecified in 9/54 studies and was less than 24 months in a further nine studies. The graphical assessment of homogeneity for the 51 comparisons of survival data is shown in Figure 1. The trials appear to be heterogeneous, and this is confirmed by the *Q*-statistic of 111.1 which, on 50 degrees of freedom, corresponds to a *P*-value of $<10^{-6}$: we can reject the null hypothesis of homogeneity among trials. This degree of inhomogeneity is unsurprising given the wide variations in eligibility criteria and times chosen for the estimation of survival.

The data for all 51 comparisons are presented in Table II. The odds ratio, rate difference, $\chi^2$ for difference in survival

**Figure 1** Scatter plot of event rates for the comparisons of survival data: neoad, (■) neoadjuvant studies; post, (□) chemotherapy given after definitive therapy; synch (◆) chemotherapy given synchronously with radiotherapy.

**Figure 2** The rate differences for the 51 comparisons of survival data. Study numbers are the reference numbers for each trial (see Appendix). ■, upper 95% confidence limit by the DerSimonian Laird method; □, lower 95% confidence limit by the DerSimonian Laird method.

between treatment and control arms and *P*-value calculated from $\chi^2$ are shown for each trial. Using $P<0.05$ as the criterion for a positive result, only nine studies were positive by both the rate difference and odds ratio methods; 39 were negative by both methods and three were positive by the odds ratio method but negative by the rate difference method. For trials defined as non-significant ($P>0.05$), the probability that the result is a false negative has been shown for a 25% relative increase in survival in the chemotherapy arm. A relative increase in survival of 25% corresponds to an increase, in absolute terms, from 40% to 50% or from 16% to 20%. Of the 42 negative comparisons, 14 had a >25% probability of being false negative and five had a probability of being false negative of >50%.

The 95% confidence limits of the rate differences are shown in Figure 2. Trials lying above the zero axis indicate possible benefit from chemotherapy; trials lying below it indicate a disadvantage from chemotherapy. Trials whose confidence limits straddle the zero axis are, by this method, non-significant at the 0.05 level of significance. Figure 3 uses a similar convention, but this time trials analysed by separate categories: neoadjuvant studies; synchronous studies using single agents; and studies using platinum/5-FU combination chemotherapy.

Table III shows the pooled estimates for odds ratio and rate difference and their confidence limits. The table also includes $\chi^2$ for difference between the control and treatment groups in terms of the end point specified, and *Q*-statistics (for homogeneity). Data are shown for survival for the whole group, and for the subgroups. Data on local control were available from 43 comparisons and data on distant metastases were available for 29 studies. These data are also shown in Table III.

The meta-analysis shows that chemotherapy produces a small, but clinically significant, improvement in survival: 6.9% with 95% confidence limits of 3.4% and 10.3%. The difference is statistically highly significant, $P<10^{-10}$. This conclusion is relatively insensitive to publication bias. Sensi-

Table II Summary of survival data for the 51 comparisons

| Trial | Type | No. of pts | Rate diff. | RD low | RD high | Odds ratio | OR low | OR high | Chi sq | P for sig. | PFN |
|---|---|---|---|---|---|---|---|---|---|---|---|
| 1 | p | 142 | 0.17 | 0.02 | 0.33 | 2.14 | 1.07 | 4.27 | 4.69 | 0.030 | |
| 4 | p | 32 | −0.31 | −0.64 | 0.02 | 0.30 | 0.08 | 1.16 | 3.04 | 0.081 | 0.021 |
| 39 | p | 229 | −0.09 | −0.21 | 0.04 | 0.69 | 0.40 | 1.17 | 1.91 | 0.167 | 0.007 |
| 48 | p | 65 | 0.02 | −0.22 | 0.26 | 1.08 | 0.40 | 2.86 | 0.02 | 0.884 | 0.468 |
| 57 | p | 46 | 0.28 | 0.04 | 0.53 | 4.62 | 1.20 | 17.85 | 4.92 | 0.026 | |
| 6 | n | 100 | −0.02 | −0.18 | 0.14 | 0.88 | 0.34 | 2.25 | 0.07 | 0.788 | 0.005 |
| 9 | n | 638 | 0.02 | −0.04 | 0.09 | 1.13 | 0.79 | 1.63 | 0.47 | 0.495 | <.001 |
| 15 | n | 78 | −0.05 | −0.27 | 0.16 | 0.81 | 0.33 | 2.00 | 0.21 | 0.644 | 0.067 |
| 17 | n | 108 | 0.02 | −0.17 | 0.20 | 1.07 | 0.50 | 2.27 | 0.03 | 0.864 | 0.309 |
| 18 | n | 187 | 0.03 | −0.10 | 0.17 | 1.15 | 0.63 | 2.12 | 0.21 | 0.644 | 0.040 |
| 24 | n | 446 | 0.08 | −0.01 | 0.17 | 1.39 | 0.95 | 2.02 | 2.92 | 0.087 | 0.103 |
| 26 | n | 75 | 0.09 | −0.07 | 0.25 | 2.06 | 0.58 | 7.36 | 1.25 | 0.264 | 0.024 |
| 27 | n | 75 | 0.15 | −0.07 | 0.37 | 1.80 | 0.73 | 4.45 | 1.63 | 0.201 | 0.886 |
| 28 | n | 218 | 0.05 | −0.08 | 0.18 | 1.23 | 0.71 | 2.12 | 0.54 | 0.464 | 0.426 |
| 29 | n | 107 | −0.03 | −0.21 | 0.16 | 0.90 | 0.42 | 1.94 | 0.08 | 0.783 | 0.065 |
| 31 | n | 36 | 0.19 | −0.08 | 0.47 | 2.56 | 0.57 | 11.44 | 1.51 | 0.218 | 0.895 |
| 32 | n | 237 | 0.09 | −0.02 | 0.20 | 1.59 | 0.88 | 2.88 | 2.39 | 0.122 | 0.030 |
| 34 | n | 59 | 0.24 | 0.01 | 0.47 | 2.83 | 0.97 | 8.26 | 3.64 | 0.057 | 0.730 |
| 35 | n | 23 | −0.01 | −0.24 | 0.22 | 0.91 | 0.05 | 15.62 | 0.00 | 0.950 | 0.045 |
| 36 | n | 60 | 0.00 | −0.25 | 0.25 | 1.00 | 0.37 | 2.73 | 0.00 | 1.000 | 0.330 |
| 37 | n | 39 | 0.02 | −0.22 | 0.26 | 1.17 | 0.23 | 5.91 | 0.04 | 0.849 | 0.058 |
| 41 | n | 158 | −0.04 | −0.18 | 0.11 | 0.85 | 0.43 | 1.67 | 0.23 | 0.629 | 0.003 |
| 45 | n | 85 | −0.02 | −0.20 | 0.17 | 0.91 | 0.34 | 2.44 | 0.03 | 0.855 | 0.018 |
| 47 | n | 68 | −0.12 | −0.35 | 0.11 | 0.62 | 0.24 | 1.60 | 0.97 | 0.320 | 0.040 |
| 49 | n | 82 | −0.02 | −0.23 | 0.19 | 0.90 | 0.37 | 2.19 | 0.05 | 0.821 | 0.093 |
| 51 | n | 332 | −0.04 | −0.15 | 0.06 | 0.84 | 0.54 | 1.30 | 0.63 | 0.427 | 0.014 |
| 52 | n | 63 | 0.09 | −0.15 | 0.33 | 1.43 | 0.53 | 3.87 | 0.49 | 0.483 | 0.834 |
| 14a | n | 292 | 0.02 | −0.09 | 0.13 | 1.10 | 0.68 | 1.78 | 0.16 | 0.686 | 0.011 |
| 14b | n | 303 | 0.10 | −0.01 | 0.21 | 1.53 | 0.96 | 2.41 | 3.25 | 0.071 | 0.269 |
| 23a | n | 40 | −0.05 | −0.31 | 0.21 | 0.76 | 0.17 | 3.27 | 0.14 | 0.708 | 0.052 |
| 23b | n | 56 | 0.00 | −0.20 | 0.20 | 1.00 | 0.26 | 3.88 | 0.00 | 1.000 | 0.017 |
| 7b | n | 34 | −0.08 | −0.46 | 0.29 | 0.71 | 0.15 | 3.31 | 0.19 | 0.660 | 0.211 |
| 2 | s | 84 | 0.32 | 0.13 | 0.52 | 3.79 | 1.59 | 9.03 | 9.04 | 0.003 | |
| 3 | s | 58 | 0.13 | −0.04 | 0.30 | 2.89 | 0.66 | 12.72 | 1.97 | 0.150 | 0.020 |
| 5 | s | 175 | 0.13 | −0.02 | 0.28 | 1.69 | 0.93 | 3.05 | 3.01 | 0.083 | 0.871 |
| 8 | s | 199 | 0.00 | −0.12 | 0.11 | 0.98 | 0.50 | 1.90 | 0.00 | 0.947 | 0.000 |
| 10 | s | 32 | 0.36 | 0.05 | 0.67 | 4.63 | 1.10 | 19.52 | 4.36 | 0.037 | |
| 11 | s | 104 | 0.19 | 0.02 | 0.37 | 2.38 | 1.05 | 5.37 | 4.33 | 0.037 | |
| 12 | s | 155 | 0.13 | −0.01 | 0.26 | 1.94 | 0.94 | 4.01 | 3.24 | 0.072 | 0.242 |
| 13 | s | 313 | 0.09 | −0.02 | 0.20 | 1.41 | 0.91 | 2.20 | 2.32 | 0.128 | 0.320 |
| 16 | s | 40 | 0.04 | −0.24 | 0.32 | 1.23 | 0.30 | 4.97 | 0.08 | 0.775 | 0.262 |
| 20 | s | 209 | 0.00 | −0.13 | 0.14 | 1.02 | 0.59 | 1.76 | 0.00 | 0.955 | 0.029 |
| 25 | s | 136 | 0.19 | 0.05 | 0.33 | 2.94 | 1.32 | 6.53 | 7.01 | 0.008 | |
| 30 | s | 157 | 0.10 | −0.05 | 0.25 | 1.56 | 0.81 | 2.99 | 1.76 | 0.185 | 0.318 |
| 40 | s | 577 | 0.28 | 0.20 | 0.36 | 3.06 | 2.21 | 4.24 | 44.87 | <.001 | |
| 44 | s | 63 | 0.23 | 0.02 | 0.45 | 2.99 | 1.01 | 8.92 | 3.88 | 0.049 | |
| 53 | s | 222 | 0.05 | −0.08 | 0.17 | 1.21 | 0.71 | 2.06 | 0.47 | 0.494 | 0.089 |
| 55 | s | 120 | 0.06 | −0.11 | 0.24 | 1.30 | 0.63 | 2.67 | 0.50 | 0.479 | 0.327 |
| 56 | s | 150 | −0.11 | −0.21 | 0.00 | 0.37 | 0.14 | 0.98 | 4.01 | 0.045 | |
| 42a | s | 116 | 0.36 | 0.20 | 0.53 | 4.34 | 2.08 | 9.05 | 15.28 | <.001 | |
| 7a | s | 20 | −0.17 | −0.59 | 0.25 | 0.49 | 0.08 | 2.95 | 0.61 | 0.435 | 0.137 |

RD, rate difference; OR, Odds ratio; low, high, 95% confidence limits; Chi sq, $\chi^2$ for significance; PFN, Probability that a trial is false negative, given a 25% relative survival benefit for chemotherapy.

Table III Summary of pooled data

| Group | No. of studies | No. of patients | Pooled RD (%) | Low (%) | High (%) | Pooled OR | Low | high | Chi squared | P | Q |
|---|---|---|---|---|---|---|---|---|---|---|---|
| All survival | 52 | 7443 | 6.5 | 3.1 | 9.9 | 1.37 | 1.24 | 1.5 | 39.6 | 1E-09 | 117 |
| All (locoregional control) | 43 | 5389 | 7.9 | 1.9 | 13.9 | 1.44 | 1.28 | 1.63 | 37.2 | 1E-08 | 256 |
| All (distant metastases) | 29 | 4883 | −1.9 | −4.8 | 1.1 | 0.79 | 0.67 | 0.93 | 8.02 | 0.02 | 64 |
| Platinum/5FU (survival) | 8 | 1636 | 10.1 | −4.7 | 25.0 | 1.56 | 0.81 | 2.99 | 4.91 | 0.025 | 11 |
| Neoadjuvant (survival) | 28 | 4141 | 3.7 | 0.9 | 6.5 | 1.2 | 1.04 | 1.35 | 6.4 | 0.011 | 20 |
| Synchronous single agent | 16 | 2506 | 12.1 | 5.0 | 19.0 | 1.77 | 1.51 | 2.1 | 54.7 | 1E-12 | 66 |

Chi squared is for significance. $Q$ is for homogeneity and is analogous to a $\chi^2$ on ($n$-1) degrees of freedom, where $n$ is the number of studies. The null hypothesis is that the trials are homogeneous. Low and high refer to the lower and upper bounds of the 95% confidence interval.

**Figure 3** ■, Upper 95% confidence limit by the DerSimonian Laird method; □, lower 95% confidence limit by the DerSimonian Laird method. **a**, Rate differences for neoadjuvant studies. **b**, Rate differences for studies of synchronous chemotherapy and radiotherapy. **c**, Rate differences for adjuvant chemotherapy with cisplatinum/5-fluorouracil.

tivity analyses show that to overturn this positive conclusion would require:

- an unreported trial containing 800 patients with 25% survival in the chemotherapy group and 75% survival in the control group.

*or*

- an unreported trial with 50% survival rate in each arm and more than 20 000 patients randomised.

Even adding 20 negative studies with survival rates of 33% in each arm and 1200 patients randomised in each trial, the overall $\chi^2$ would still be 9.71 ($P<0.005$). No single study was unduly influential. Eliminating significant studies in sequence did not affect the conclusions. For example, even if the 11 most significant studies were eliminated completely, the overall $\chi^2$ was still 5.29 ($P=0.021$).

The results from the sensitivity analyses dealing with possible bias in data publication and extraction are shown in Figure 4. The robustness of the conclusion is sensitive to this type of bias. A constant bias of 5% produces results similar to a bias varying randomly for each trial between 0 and 10%; this again suggests that no one trial is unduly influential.

The subgroup analyses suggest that single-agent chemotherapy given with radiotherapy is particularly effective – rate difference 13.7% (95% CI 6.1–21.3%) – but neoadjuvant chemotherapy is somewhat less effective – rate difference 3.9% (95% CI 1.1–6.7%). Platinum/5-FU regimens do not appear to be outstandingly effective – rate difference 5.4% (95% CI 0.1–10%). The data on local control are consistent

**Figure 4** Sensitivity analyses of bias in data presentation and extraction. The method for correcting for possible bias is described in the text. **a**, The rate difference method, with 95% confidence intervals (DerSimonian and Laird). **b**, The odds ratio method, with 95% confidence intervals (Peto).

with the data on survival. The data on distant metastases are, in this respect, less consistent.

### Discussion

This overview of trials of adjuvant chemotherapy in head and neck cancer suggests that chemotherapy might improve survival and that this improvement is more apparent for single-agent chemotherapy given synchronously with radiotherapy. Since two previous meta-analyses (Stell and Rawson, 1990; Stell, 1992) failed to show benefit from chemotherapy, the discrepancies between these previous analyses and the current results must be explained. Stell and Rawson's first analysis (1990) included 23 trials, and the updated analysis added five newer trials to give a total of 28 trials (Stell, 1992). The recent flurry of trial publication means that there are now many more trials for analysis: 51 comparisons for survival effect. The second overview was not particularly robust: the $z$-value for overall survival was 1.24 ($P>0.05$). It would only be necessary to add a single trial with a total of 380 patients randomised, with survival rates of 47.3% in the chemotherapy arm and 34.2% in the control arm, to convert this non-significant $z$-value to a significant one.

Cumulative meta-analyses, and the current study could be regarded as the third in a sequence for head and neck cancer, can be useful for the prompt detection of therapeutic advances. Experience from trials of treatment for myocardial infarction showed that, although early overviews were negative, the accumulation of evidence eventually favoured active therapy (Antman *et al*., 1992; Lau *et al*., 1992).

The main disadvantage of the present analysis is that it is based upon the published literature rather than upon data from individual patients. This raises problems with the assessment of event rates (Stewart and Parmar, 1993). The inability to use a constant time point for survival, for example, introduces potentially serious bias since the survival at arbitrary time points does not, and cannot, represent the overall shape of the survival curve. The sensitivity analyses clearly show that the overall conclusion of this overview is sensitive to this type of bias. The only solution is to perform a per-patient analysis, and such a study is currently under way (MKB Parmar, 1994, personal communication). Unfortunately, it will be at least 2 years until the results are published; in the meantime literature-based analysis, with all its imperfections, will have to suffice.

The present overview suggests that the largest gains, in terms of survival, may be obtained by using chemotherapy synchronously with radiotherapy. The demonstration that gains from neoadjuvant therapy are relatively modest compared with the benefits from synchronous therapy is provocative and, if true, would require an explanation consistent with the basic biology of squamous carcinoma of the head and neck. Squamous carcinomas of the head and neck have high cell loss factors: 90% of cells produced by mitosis of clonogenic cells may be lost through exfoliation and migration. Relatively modest killing of clonogens will, through the effects of cell loss, produce rapid shrinkage of tumour. This rapid regression, is, however, virtually an epiphenomenon – albeit a gratifying one.

The ultimate outcome is dictated by those clonogenic cells which are not lost and, in particular, their resistance to therapy. Because of cell loss, a clinically apparent tumour is genetically old, a 2 cm squamous cell carcinoma of the head and neck is perhaps 600–1000 generations old. In the absence of cell loss it would take only 30–40 generations to reach this size. The chance of a mutation emerging that confers drug resistance increases with each generation. There is a high probability that, at diagnosis, even small tumours of the head and neck will contain clonogenic cells which are, *de novo*, resistant to cytotoxic drugs. Cell loss can therefore explain both the initial responsiveness and the ultimate resistance to chemotherapy of these tumours.

Accelerated repopulation of clonogenic cells in tumours may compromise the effectiveness of radiotherapy for head and neck cancers (Withers *et al.*, 1988). Neoadjuvant chemotherapy, by providing the stimulus for such repopulation several weeks before the start of radiotherapy, might exacerbate this problem. With synchronous chemotherapy, the problem of such treatment-induced perturbations does not apply.

The data on the effects of chemotherapy upon distant metastasis are conflicting. This partly reflects the fact that distant metastases are an uncommon cause of treatment failure in head and neck cancer. The majority of patients who die do so from local regional failure. The inability of chemotherapy to prevent distant metastasis may therefore be more apparent than real.

An overview has two main purposes: firstly to suggest what, on the basis of data from clinical trials, might be defined as reasonable current practice; secondly, to provide a stimulus to further studies. Primary treatment with chemotherapy may provide useful relief of symptoms in patients treated palliatively, but there is little justification for the routine use of neoadjuvant chemotherapy in head and neck cancer. The claim, from the Veterans Administration study (The Department of Veterans Affairs Laryngeal Cancer Study Group, 1991), that neoadjuvant chemotherapy offers the possibility of avoiding mutilating surgery in head and neck cancer is controversial since that study, by virtue of its design, was unable to provide any evidence that chemotherapy plus radiotherapy was any better than radiotherapy alone.

The data presented here suggest that we might put less effort into neoadjuvant studies and return to a more detailed investigation of the effectiveness of single-agent chemotherapy given synchronously with radiotherapy. Such treatment is simple and inexpensive. The survival benefit may be genuine: the next questions are what are the costs of such benefit in terms of excess morbidity and which is the best drug to use? Future trials will need to collect adequate data, both objective and subjective, on the toxicity of treatment. Radiation dose may also be important. It is essential that trials of synchronous chemotherapy report the radiation doses actually given, not simply those that were intended. If synchronous chemotherapy increases acute morbidity and necessitates the attentuation or curtailment of radiation therapy, then there may be little overall gain. Trials designed to answer these important questions need not be complex, nor should their entry criteria be too restrictive. Large simple studies are now required (Peto and Easton, 1989) to define more precisely the contribution of synchronous chemotherapy to the radiotherapeutic management of head and neck cancer.

**Acknowledgements**
I would like to thank Dr MKB Parmar of the MRC Clinical Trials Unit, Cambridge, for his constructive criticism and advice on earlier drafts of this manuscript.

**References**

ANTMAN EM, LAU J, KUPELNICK B, MOSTELLER F AND CHALMERS TC. (1992). A comparison of results of meta-analyses of randomized control trials and the recommendations of clinical experts. *JAMA*, **268**, 240–248.

CACHIN Y. (1978). Cancer of the head and neck. In *Randomized Trials in Cancer: a Critical Review by Sites*, MJ (ed.) pp. 331–338. Raven Press: New York.

DERSIMONIAN R AND LAIRD N. (1986). Meta-analysis in clinical trials. *Controlled Clin. Trials*, **7**, 177–188.

DETSKY AS AND SACKETT DL. (1985). When was a 'negative' clinical trial big enough? How many patients you needed depends upon what you found. *Arch. Int. Med.*, **145**, 709–712.

DIMERY IW AND HONG WK. (1993). Overview of combined modality therapies for head and neck cancer. *J. Natl Cancer Inst.*, **85**, 95–111.

DODION P, ANDRY G AND BALIKDJIAN D. (1986). Head and Neck Cancer. In *Randomized Trials in Cancer: a Critical Review by Sites*, Staquet MJ and Slevin ML (eds) pp. 525–547. Raven Press: New York.

EARLY BREAST CANCER TRIALISTS' COLLABORATIVE GROUP (1990). *Treatment of Early Breast Cancer*, Vol. 1, *worldwide evidence 1985–1990*. Oxford University Press: Oxford.

LAU J, ANTMAN EM, JIMENEZ-SILVA J, KUPELNICK B, MOSTELLER F AND CHALMERS TC. (1992). Cumulative meta-analysis of therapeutic trials for myocardial infarction. *N. Engl. J. Med.*, **327**, 248–254.

PETO J AND EASTON D. (1989). Cancer treatment trials – past failures, current progress and future prospects. *Cancer Surv.*, **8**, 511–533.

STELL PM. (1992). Adjuvant chemotherapy for head and neck cancer. *Semin. Radiat. Oncol.*, **2**, 195–205.

STELL PM AND RAWSON NSB. (1990). Adjuvant chemotherapy in head and neck cancer. *Br. J. Cancer*, **61**, 779–787.

STEWART LA AND PARMAR MKB. (1993). Meta-analysis of the literature or individual patient data: is there a difference? *Lancet*, **341**, 418–422.

TANNOCK IF AND BROWMAN GP. (1986). Lack of evidence for a role of chemotherapy in the routine management of locally advanced head and neck cancer. *J. Clin. Oncol.*, **4**, 1121–1126.

TAYLOR SG. (1987). Why has so much chemotherapy done so litte in head and neck cancer? *J. Clin. Oncol.*, **5**, 1–3.

THE DEPARTMENT OF VETERANS AFFAIRS LARYNGEAL CANCER STUDY GROUP (1991). Induction chemotherapy plus radiation compared with surgery plus radiation in patients with advanced laryngeal cancer. *N. Engl. J. Med.*, **324**, 1685–1690.

WITHERS HR, TAYLOR JMG AND MACIEJEWSKI B. (1988). The hazard of accelerated tumor clonogen repopulation during radiotherapy. *Acta Oncol.*, **27**, 131–146.

**Appendix: List of trials analysed with reference numbers**

1 ARCANGELI G, NERVI C, RIGHINI R, CRETON G, ALESSANDRA MIRRI M AND GUERRA A. (1983). Combined radiation and drugs: the effect of intra-arterial chemotherapy followed by radiotherapy in head and neck cancer. *Radiother. Oncol.*, **1**, 101-107.

2 BACHAUD J, DAVID J, BOUSSIN G AND DALY N. (1991). Combined postoperative radiotherapy and weekly cisplatin infusion for locally advanced squamous cell carcinoma of the head and neck: preliminary report of a randomized trial. *Int. J. Radiat. Oncol. Biol. Phys.*, **20**, 243-246.

3 BEZWODA WR, DEMOOR NG AND DERMAN DP. (1979). Treatment of advanced head and neck cancer by means of radiation therapy plus chemotherapy – a randomized trial. *Medical Pediatr. Oncol.*, **6**, 353-358.

4 BITTER K. (1981). Postoperative chemotherapy versus postoperative Cobalt 60 radiation in patients with advanced oral carcinoma – report on a randomized study. *Head Neck Surg.*, **3**, 264.

5 BROWMAN GP, HODSON I, LEVINE MN, SATHYA J, RUSSELL R, CRIPPS C, EAPEN L, GIRARD A AND PANETTA D. (1993). Placebo-controlled randomized trial of intravenous infusional 5-fluorouracil (FU) concurrent with standard radiotherapy (RT) in Stages III and IV head and neck cancer (HNC). *ASCO Proc.*, **12**, 277-891A.

6 BRUNIN F, RODRIGUEZ J, JAULERRY C, JOUVE M, PONTVERT D, POINT D, MOSSERI V, POUILLART P, ASSELAIN B, BRUGERE J AND BATAINI JP. (1989). Induction chemotherapy in advanced head and neck cancer. Preliminary results of a randomized study. *Acta Oncol.*, **28**, 61-65.

7 DOGGETT RLS, BAGSHAW MA AND KAPLAN HS. (1967). Combined therapy using chemotherapeutic agents and radiotherapy. In *Modern Trends in Radiotherapy*, Deeley TJ and Wood CAP (eds) pp. 107-131. Butterworths: London.
7a synchronous chemotherapy
7b neoadjuvant chemotherapy

8 ESCHWEGE F, SANCHO-GARNIER H, GERARD JP, MADELAIN M, DESAULTY A, JORTAY A AND CACHIN Y. (1988). Ten year results of randomized trial comparing radiotherapy and concomitant bleomycin to radiotherapy alone in epidermoid carcinomas of the oropharynx: experience of the European Organization for the Research and Treatment of Cancer. *Natl Cancer Instmonogr.*, **6**, 275-278.

9 FAZEKAS JT, SOMMER C AND KRAMER S. (1980). Adjuvant intravenous methotrexate or definitive radiotherapy alone for advanced squamous cancers of the oral cavity, oropharynx, supraglottic larynx or hypopharynx. Concluding report of an RTOG randomozed trial on 638 patients. *Int. J. Radiat. Oncol. Biol. Phys.*, **6**, 533-541.

10 FLETCHER GH, SUIT HD, HOWE CD, SAMUELS M, JESSE RH AND VILLAREAL RU. (1963). Clinical method of testing radiation-sensitizing agents in squamous cell carcinoma. *Cancer*, **16**, 355-363.

11 FU KK, PHILLIPS TL, SILVERBERG IJ, JACOBS C, GOFFINET DR, CHUN C, FRIEDMAN MA, KOHLER M, MCWHIRTER K AND CARTER SK. (1987). Combined radiotherapy and chemotherapy with bleomycin and methotrexate for advanced inoperable head and neck cancer: update of Northern California Oncology Group randomized trial. *J. Clin. Oncol.*, **5**, 1410-1418.

12 GOLLIN FF, ANSFIELD FJ, BRANDENBURG JH, RAMIREZ G AND VERMUND H. (1972). Combined therapy in advanced head and neck cancer: a randomized study. *Am. J. Roentgenol. Radium Ther. Nucl. Med.*, **114**, 83-88.

13 GUPTA NK, POINTON RCS AND WILKINSON PM. (1987). A randomized clinical trail to contrast radiotherapy with radiotherapy and methotrexate given synchronously in head and neck cancer. *Clin. Radiol.*, **38**, 575-581.

14 HEAD AND NECK CONTRACTS PROGRAM (1987). Adjuvant chemotherapy for advanced head and neck squamous carcinoma. Final report of the head and neck contracts program. *Cancer*, **60**, 301-310.
14a neoadjuvant *vs* standard therapy
14b neoadjuvant + maintenance *vs* standard therapy

15 HOLOYE PY, GROSSMAN TW, TOOHILL RJ, KUN LE, BYHARDT RW, DUNCAVAGE JA, TEPLIN RW, RITCH PS, HOFFMAN RG AND MALIN TA. (1985). Randomized study of adjuvant chemotherapy for head and neck cancer. *Otolaryngol. Head Neck Surg.*, **93**, 712-717.

16 HUSSEY DH AND ABRAMS JP. (1975). Combined therapy in advanced head and neck cancer: hydroxyurea and radiotherapy. *Prog. Clin. Cancer*, **6**, 79-86.

17 JAULERRY C, RODRIGUEZ J, BRUNIN F, JOUVE M, MOSSERI V, POINT D, PONTVERT D, VALIDIRE P, ZAFRANI B, BLASZKA B, ASSELAIN B, POUILLART P AND BRUGERE J. (1992). Induction chemotherapy in advanced head and neck tumors: results of two randomized trials. *Int. J. Radiat. Oncol. Biol. Phys.*, **23**, 483-489.

18 JORTAY A, DEMARD F, DALESIO O, BLANCHET C, DESAULTY A, GEHANNO C, LEFEBRE JL, MOLINARI R, TRAISSAC L, DEHESDIN M & KIRKPATRICK A. (1990). A randomized EORTC study on the effect of preoperative polychemotherapy in pyriform sinus carcinoma treated by pharyngolaryngectomy and irradiation: results from 5 to 10 years. *Acta. Chir. Belg.*, **90**, 115-122.

19 KAPSTAD B, BANG G, RENNAES S AND DAHLER A. (1978). Combined preoperative treatment with cobalt and bleomycin in patients with head and neck carcinoma – a controlled clinical study. *Int. J. Radiat. Oncol. Biol. Phys.*, **4**, 85-89.

20 KEANE TJ, CUMMINGS BJ, O'SULLIVAN B, PAYNE D, RAWLINSON E, MACKENZIE R, DANJOUX C AND HODSON I. (1993). A randomized trial of radiation therapy compared to split course radiation therapy combined with mitomycin C and 5 fluorouracil as initial treatment for advanced laryngeal and hypopharyngeal squamous carcinoma. *Int. J. Radiat. Oncol. Biol. Phys.*, **25**, 613-618.

21 KEEGAN P, PILLSBURY HR, WEISSLER M, FRY T AND ROSEMAN JR. (1988). Simultaneous cisplatinum-5fu and radiotherapy vs radiotherapy alone in advanced squamous carcinoma of the head and neck. *ASCO Proc.*, **7**, 157-609A.

22 KLIGERMAN MM, HELLMAN S, VON ESSEN CF AND BERTINO JR. (1966). Sequential chemotherapy and radiotherapy: preliminary results of a clinical trial with methotrexate in head and neck cancer. *Radiology*, **86**, 247-250.

23 KNOWLTON AH, PERCAPIO B, BOBROW S AND FISHER JJ. (1975). Methotrexate and radiation therapy in the treatment of advanced head and neck tumors. *Radiology*, **116**, 709-712.
23a high-dose methotrexate
23b low-dose methotrexate

24 LARAMORE GE, SCOTT CB, AL SARRAF M, HASELOW RE, ERVIN TJ, WHEELER R, JACOBS JR, SCHULLER DE, GAHBAUER RA, SCHWADE JG AND CAMPBELL BH. (1992). Adjuvant chemotherapy for resectable squamous cell carcinomas of the head and neck: report on intergroup study 0034. *Int. J. Radiat. Oncol. Biol. Phys.*, **23**, 705-713.

25 LO TCM, WILEY AL, ANSFIELD FJ, BRANDENDBURG JH, DAVIS HL, GOLLIN FF, JONSON RO, RAMIREZ G AND VERMUND H. (1976). Combined radiation therapy and 5-fluorouracil for advanced squamous cell carcinoma of the oral cavity and oropharynx: a randomized study. *Am. J. Roentgenol. Radium Ther. Nucl. Med.*, **126**, 229-235.

26 LUSTIG RA, DEMARE PA AND KRAMER S. (1976). Adjuvant methotrexate in the radiotherapeutic management of advanced tumors of the head and neck. *Cancer*, **37**, 2703-2708.

27 MARTIN M, HAZAN A, VERGNES L, PEYTRAL C, MAZERON JJ, SENECHAUT JP, LELIÈIVRE G AND PEYNÈGRE R. (1990). Randomized study of 5 Fluorouracil and cis-platin as neoadjuvant therapy in head and neck cancer: a preliminary report. *Int. J. Radiat. Oncol. Biol. Phys.*, **19**, 973-975.

28 MARTIN M, LEIÈVRE G, GEHANNO C, DEPONDT J, GUERRIER B, PEYTRAL C, HAZAN A, DUBREUIL P, MARGOTTON A AND PELLAE-COSSET B. (1992). Induction carboplatin (CBDCA) and 5-fluorouracil (5-FU) treatment versus no chemotherapy before locoregional treatment for oro and pharyngolaryngeal cancers: preliminary results of a randomized study. *ASCO Proc.*, **11**, 240.

29 MARTIN M, MAZERON JJ, BRUN B, VERGNES L, LELIEVRE G, FEUILLADE F, JUVANON JM, HADDAD E, SOUCHAL DELACOUR I, PEYNEGRE R AND PIERQUIN B. (1988). Neoadjuvant polychemotherapy of head and neck cancer: results of a randomized study. *ASCO Proc.*, **7**, 152.

30 MERLANO M, VITALE V, ROSSO R, BENASSO M, CORVO R, CAVALLARI M, SANGUINETI G, BACIGALUPO A, BADELLINO F, MARGARINO G, BREMA F AND PASTORINO G. (1992). Treatment of advanced squamous-cell carcinoma of the head and neck with alternating chemotherapy and radiotherapy. *N. Engl. J. Med.*, **327**, 1115-1121.

31 NISSENBAUM M, BROWDE S, BEZWODA WR, DEMOOR NG AND DERMAN DP. (1984). Treatment of advanced head and neck cancer: multiple daily dose fractionated radiation therapy and sequential multimodal treatment approach. *Med. Pediatr. Oncol.*, **12**, 204-208.

32 PACCAGNELLA A, ORLANDO A, MARCHIORI C, ZORAT PL, CHIARION-SILENI V, JIRILLO A, TOMIO L, FILA G, FEDE A, BARI M, GAVA A, PAPPAGALLO GL AND FIORENTINO MV. (1993). A phase III trial of neoadjuvant chemotherapy in head and neck cancer. *ASCO Proc.*, **12**, 894A.

34 PEARLMAN NW, JOHNSON FB, BRAUN TJ, KENNAUGH RC, SPOFFORD BF, BORLASE BC, MEYER TJ, STIEGMANN GV AND MEYERS AD. (1985). A prospective study of preoperative chemotherapy and split-course irradiation for locally advanced or recurrent oral-pharyngeal squamous carcinoma. *Am. J. Clin. Oncol. (CCT)*, **8**, 490-496.

35 PETROVICH Z, BLOCK J, KUISK H, MACKINTOSH R, CASCIATO D, JOSE L AND BARTON R. (1981). A randomized comparison of radiotherapy with a radiotherapy- chemotherapy combination in stage IV carcinoma of the head and neck. *Cancer*, **47**, 2259-2264.

36 RENTSCHLER RE, WILBUR DW, PETTI GH, CHONKICH GD, HILLIARD DA, CAMACHO ES AND THORPE RB. (1987). Adjuvant methotrexate escalated to toxicity for resectable Stage III and IV squamous head and neck carcinomas - a prospective randomized study. *J. Clin. Oncol.*, **5**, 278-285.

37 RICHARD JM, SANCHO H, LEPINTRE Y, RODARY J AND PIERQUIN B. (1974). Intra-arterial methotrexate chemotherapy and telecobalt therapy in cancer of the oral cavity and oropharynx. *Cancer*, **34**, 491-496.

38 RICHARDS GJ AND CHAMBERS RG. (1969). Hydroxyurea: a radiosensitizer in the treatment of neoplasms of the head and neck. *Am. J. Roentgenol. Radium Ther. Nucl. Med.*, **105**, 555-565.

39 ROSSI A, MOLINARI R, BORACCHI P, DEL VECCHIO M, MARUBINI E, NAVA M, MORANDI L, ZUCALI R, PILOTTI S, GRANDI C, AMBROSINI G, CELLINI N, CHIAVACCI A, COLOMBO A, DAL FIOR S, DE MARIA D, FELCI U, GABRIELE P, LADDAGA M, MAGNO L, MARZIANO C, OLMI P, PRINO A, RONCORONI L, TORRETTA A, ZAMPI G, ZORAT PL AND DE PALO G. (1988). Adjuvant chemotherapy with Vincristine, Cyclophosphamide and Doxorubicin after radiotherapy in loco-regional nasopharyngeal cancer: results of a 4-year multicenter randomized study. *J. Clin. Oncol.*, **6**, 1401-1410.

40 SANCHIZ F, MILLA A, TORNER J, BONET F, ARTOLA N, CARRENO L, MOYA LM, RIERA D, RIPOL S AND CIRERA L. (1990). Single fraction per day versus two fractions per day versus radiochemotherapy in the treatment of head and neck cancer. *Int. J. Radiat. Oncol. Biol. Phys.*, **19**, 1347-1350.

41 SCHULLER DE, METCH B, MATTOX D, STEIN DW AND McCRAKEN JD. (1988). Preoperative chemotherapy in advanced resectable head and neck cancer: final report of the southwest oncology group. *Laryngoscope*, **98**, 1205-1211.

42 SHANTA V AND KRISHNAMURTHI S. (1980). Combined bleomycin and radiotherapy in oral cancer. *Clin. Radiol.*, **31**, 617-620.

42a sychronous

42b neoadjuvant

44 SHIGEMATSU Y, FUCHIHATA H, MAKINO T AND INOUE T. (1973). Radiotherapy with reduced fraction in head and neck cancer with special reference to hyperbaric oxygen radiotherapy in maxillary sinus carcinoma (a controlled study). In *Fraction Size in Radiobiology and Radiotherapy*, Sugahara T, Scott OCA and Revesz L (eds) pp. 180-187. Williams & Wilkins: Baltimore.

45 SIODLAK MZ, DALBY JE, BRADLEY PJ, CAMPBELL JB, STRICKLAND P, FRASER JG, WILLATT DJ, FLOOD LM AND STELL PM. (1989). Induction VBM plus radiotherapy versus radiotherapy alone for advanced head and neck cancer: long term results. *Clin. Otolaryngol.*, **14**, 17-22.

47 STOLWIJK C, WAGENER DJT, VAN DEN BROEK P, LEVENDAG PC, KAZEM I, BRUASET I AND DE MULDER PHM. (1985). Randomized neo-adjuvant chemotherapy trial for advanced head and neck cancer. *Neth. J. Med.*, **28**, 347-351.

48 SZPIRGLAS H, CHASTANG C AND BERTRAND JC. (1978). Adjuvant treatment of tongue and floor of mouth cancers. *Recent Results Cancer Res.*, **68**, 309-317.

49 TAYLOR SG, APPLEBAUM E, SHOWEL JL, NORUSIS M, HOLINGER LD, HUTCHINSON JC, MURTHY AK AND CALDARELLI DD. (1985). A randomized trial of adjuvant chemotherapy in head and neck cancer. *J. Clin Oncol.*, **3**, 672-679.

50 TEJADA F AND CHANDLER JR. (1982). Combined therapy for stage III and IV head and neck cancer (H&N). *ASCO Proc.*, **1**, 199.

51 THE DEPARTMENT OF VETERANS AFFAIRS LARYNGEAL CANCER STUDY GROUP (1991). Induction chemotherapy plus radiation compared with surgery plus radiation in patients with advanced laryngeal cancer. *N. Engl. J. Med.*, **324**, 1685-1690.

52 TOOHILL RJ, ANDERSON T, BYHARDT RW, COX JD, DUNCAVAGE JA, GROSSMAN TW, HAAS CD, HAAS JS, HARTZ AJ, LIBNOCH JA, MALIN TC, RITCH PS AND WILSON JF. (1987). Cisplatin and fluorouracil as neoadjuvant therapy in head and neck cancer. *Arch. Otolaryngol. Head Neck Surg.*, **113**, 758-761.

53 VERMUND H, KAALHUS O, WINTHER F, TRAUSJO J, THORUD E AND HARANG R. (1985). Bleomycin and radiation therapy in squamous cell carcinoma of the upper aero-digestive tract: a phase III clinical trial. *Int. J. Radiat. Oncol. Biol. Phys.*, **11**, 1877-1886.

54 VON ESSEN CF, JOSEPH LBM, SIMON GT, SINGH AD AND SINGH SP. (1968). Sequential chemotherapy and radiation therapy of buccal mucosa carcinoma in South India. *Am. J. Roentgenol. Radium Ther. Nucl. Med.*, **102**, 530-540.

55 WEISSBERG JB, SON YH, PAPAC RJ, SASAKI C, FISCHER DB, LAWRENCE R, ROCKWELL S, SARTORELLI AC AND FISCHER JJ. (1989). Randomized clinical trial of Mitomycin C as an adjunct to radiation therapy in head and neck cancer. *Int. J. Radiat. Oncol. Biol. Phys.*, **17**, 3-9.

56 STEFANI S AND CHUNG TS. (1980). Hydroxyurea and radiotherapy in head and neck cancer - long term results of a double blind prospective study. *Int. J. Radiat. Oncol. Biol. Phys.*, **6**, 1398-190A.

57 ERVIN TJ, CLARK JR, WEICHSELBAUM RR, FALLON BG, MILLER D, FABIAN RL, POSNER MR, NORRIS CM, TUTTLE SA, SCHOENFELD DA, PRICE KN AND FREI E. (1987). An analysis of induction and adjuvant chemotherapy in the multidisciplinary treatment of squamous-cell carcinoma of the head and neck. *J. Clin. Oncol.*, **5**, 10-20.